# China’s global engagement to fight the novel coronavirus pandemic

**DOI:** 10.1186/s41256-020-00172-1

**Published:** 2020-10-16

**Authors:** Wei Song

**Affiliations:** CAITEC International Development Cooperation Institute, No. 28 Donghouxiang, Anwai, Beijing, 100710 China

**Keywords:** China, Official development aid, COVID-19, Pandemic

## Abstract

The world is confronted by the current pandemic of coronavirus disease 2019 (COVID-19), which is a common threat to the whole of humanity. In the process of fighting COVID-19 domestically, China had attached great importance to international cooperation, such as the sharing of information on the pandemic with the international community, providing bilateral and multilateral assistance to other affected countries, etc. However, due to the severity of this pandemic, global solidarity is necessary to conquer it, and to improve global public health governance.

## Background

The novel coronavirus disease (COVID-19) first broke out in Wuhan in December 2019. Facing this unknown infectious disease, the Chinese government took preventative measures immediately, including implementing a nationwide quarantine, offering medical support and mobilizing resources from all over the country, enhancing public education, strengthening individual protection, medical isolation, controlling population mobility, and reducing gatherings [1]. Furthermore, the government quickly formulated the Law on ‘Punishing Legally the Crimes of Violating the Prevention of Novel Coronavirus Infected Pneumonia’ [2]. These measures are not only directly associated with China’s overall containment of the epidemic [3], but also contribute valuable experience for the international community in its fight against it. And during this process, China has received material assistance and moral support from many countries, such as Iran, Israel, Pakistan, South Korea, Japan, Cuba, Russia and some European countries. In return, China also actively provided assistance to the international community, including medical materials and knowledge-sharing. China’s commitment has been highly recognized and appreciated by many countries, such as Italy and Serbia.

Moreover, the emergence of COVID-19 coincided with the largest annual human migration in the world, i.e., the Spring Festival travel season, which brought about a rapid national and global spread of the virus [4]. The first 100 thousand cases took 67 days, the second 100 thousand cases took 11 days, and the third 100 thousand cases took only 4 days. The pandemic seriously has threatened the lives and health of people all over the world, exerting a deep impact on the global political, economic and social map. Against this backdrop, the purpose of this commentary is to analyze China’s global engagement in the fight against COVID-19, which not only is conducive to a more effective fight against the pandemic, but also illuminates the improvement in global public governance.

## China’s global engagement

In the era of globalization, the interests of human societies are highly integrated. As Chinese President Xi Jinping put it, viruses don’t respect borders, and pandemics don’t respect races. The outbreak of COVID-19 has once again demonstrated that mankind is a community of shared destiny. China has continued to firmly adhere to the concept of a community of shared destiny, working together with people of all countries to strive for a final victory in the fight.

### Exchanging views at high level

Chinese leaders have attached great importance to international cooperation concerning anti-pandemic policy, even as they fight COVID-19 domestically. Up to mid-May, President Xi Jinping had striven to exchange views with nearly 50 foreign leaders and heads of international organizations in 2 months through “telephone diplomacy” or face to face discussion. On March 26, he actively participated in the G20 special summit on the epidemic, pointing out that the international community should step up its efforts to effectively carry out joint prevention and control measures.. In addition, President Xi Jinping declared a package of humanitarian and development assistance to the Least Developed Countries and other countries seriously infected at the opening ceremony of the World Health Organization’s 73rd World Health Assembly.

### Knowledge-sharing

China actively and generously shared information on the pandemic with the international community. Thanks to China’s advantage in information communications technology and in innovative technology enterprises, China developed a relatively complete public online reporting system within a very short time [5]. China built an academic sharing platform and a communication mechanism to share genomic information and promote capacity building. In point of fact, it is the right choice to carry out international cooperation and concentrate human wisdom on vaccine research and development, as challenges abound and uncertainties lie ahead. Now China is endeavoring to develop vaccines following five technological routes: inactivated vaccines, recombinant protein vaccines, live attenuated influenza vaccines, adenovirus vaccines, and nucleic acid-based vaccines, and all of which involve international cooperation and are open to the international community. With knowledge sharing, an increasing number of countries switched to more stringent public health strategies including city lockdowns and mandatory quarantines [6]. And lockdown has now been implemented in Europe [7]. In addition, universities and hospitals actively participated in experience sharing. The Chinese diaspora, especially students overseas, was also a crucial channel for sharing and spreading related experiences, as they acquired relevant information on prevention and control measures from home and then shared it with people around them.

### Bilateral assistance

China actively provided assistance to other affected countries, meanwhile fighting against the domestic epidemic, including providing funds and medical materials, sending medical teams and so forth. Although the masks and medicines in China were at the time in short supply, the Chinese government put a premium on supporting the export of medical materials, and welcomed export enterprises to organize the external supply of masks and other medical materials, so as to make due contributions to global epidemic prevention in a practical manner. Table 1 reflects the bilateral support China has provided to global society.

**Table 1:** China’s Official Development Assistance to Global Anti-Pandemic Efforts

Material-supplying	Assisted the WHO in purchasing personal protective equipment and establishing reserve centers of supplies in China; Participated in the WHO’s “Access to COVID-19 Tools (act) Accelerator” initiative, aiming to speed up the development, production and equitable distribution of new tools; Exported protective materials to 200 countries and regions, among which there were more than 70.6 billion masks, 340 million protective suits, 115 million pairs of goggles, 96,700 ventilators, 225 million test kits, and 40.29 million infrared thermometers.
Cash support	Two batches of cash support totaling $50 million to the WHO; Helped WHO’s COVID-19 Solidarity Response Fund to raise funds in China; $2 billion to the international community within 2 years;
Capacity building	29 medical expert teams were sent to 27 countries; Medical teams stationed in 56 countries supported the local fight, and provided counseling and health information to local people and overseas Chinese, organizing over 400 online and offline training sessions;

Statistics at: Full Text: Fighting COVID-19: China in Action, The State Council Information Office of the People’s Republic of China, June 2020. Available at: http://www.scio.gov.cn/zfbps/32832/Document/1681809/1681809.htm

President Xi Jinping’s speech at the opening ceremony of the World Health Organization’s 73rd World Health Assembly, Xinhua Net, May 18, 2020. Available at: http://m.xinhuanet.com/2020-05/18/c_1126001593.htm

### Multilateral assistance

China has actively participated in international cooperation through multilateral channels: it has contributed to the World Health Organization, the United Nations, and the African Union; meanwhile, China has also donated $55 million to the WHO. Furthermore, at the opening ceremony of the video conference of the 73rd Assembly of the WHO, China’s President Xi Jinping promised to provide $2 billion within 2 years to support countries affected by the pandemic, especially developing countries, in order to help them recover economically and socially. Finally, once the vaccine is developed, China has committed to provide it to the international community as a global public product. Of note, the World Food Program (WFP), with the support of the Chinese government, established the UN Global Humanitarian Response hub in China, and the first batch of anti-pandemic supplies arrived in the warehouse hub in Guangzhou in April 30, and was transported to other emergency points identified by the United Nations or directly to the countries and regions affected by COVID-19. With its leading manufacturing industry, complete supply chain, and technological innovation, China holds unique advantages in setting up an emergency hub.The above Table 1 also reflects the multilateral support China has provided to the global society.

### Public-private partnership

China is striving to mobilize various institutions and parties to support the global anti-pandemic efforts by actively promoting the Public-Private Partnership modality. In March, under the guidance of the International Liaison Department of the Central Committee of the CPC, the China NGO Network for International Exchanges (CNIE), China’s largest association of international exchange-oriented social organizations, launched the joint action “Silk Road and One Family” to fight against the pandemic, calling on non-governmental actors to assist countries in need. Under the framework of the joint action, China’s non-governmental forces have carried out various forms of anti-pandemic cooperation with more than 50 countries, including material-supplying, experience-sharing and volunteer-dispatching, with this foreign assistance volume totaling more than 25 million U.S. dollars.

## Future directions

Given the existential challenge posed by COVID-19, international cooperation is imperative to our very survival as a species. As the first priority lies in addressing the humanitarian challenge through proper preventive measures to stop its spread, as well as finding curative measures to develop a vaccine [8], it is essential to strengthen global solidarity to cooperate in a bid to conquer the pandemic, and to improve global public health governance. Although data shows that China has brought the infection rate under control, epidemiologists have warned that the pandemic may bounce back in a context where officials relax restrictions and more people return to work. And further efforts are required to determine how to strike a balance between the expected positive effect on public health and the negative impact on freedom of movement, the economy, and society at large [9]. Therefore, cooperation between major countries, especially China and the other major world power, the United States, is still the fundamental way to effectively fight against COVID-19.

As China’s Foreign Minister Wang Yi put it, COVID-19 is a common enemy for both China and United States; therefore, mutual help and support should be a common goal of the two peoples and governments. And some governments at the state level and NGOs from the United States are also actively promoting Sino-US cooperation at present. Given the structure of the current world system it is impossible for single countries to fight COVID-19 by themselves. COVID-19 will test our capacity as a species to exert effective global health governance.

## Abbreviations

COVID-19: Coronavirus disease 2019
BRI: Belt and Road Initiative
WHO: World Health Organization
